# Ecological Niche Modelling of Bank Voles in Western Europe

**DOI:** 10.3390/ijerph10020499

**Published:** 2013-01-28

**Authors:** Sara Amirpour Haredasht, Miguel Barrios, Jamshid Farifteh, Piet Maes, Jan Clement, Willem W. Verstraeten, Katrien Tersago, Marc Van Ranst, Pol Coppin, Daniel Berckmans, Jean-Marie Aerts

**Affiliations:** 1 Measure, Model & Manage Bioresponses (M3-BIORES), Biosystems Department, KU Leuven, Kasteelpark Arenberg 30, Leuven B-3001, Belgium; E-Mails: sara.amirpourharedasht@biw.kuleuven.be (S.A.H.); daniel.berckmans@biw.kuleuven.be (D.B.); 2 M3-BIORES, Biosystems Department, KU Leuven, Willem de Croylaan 34, Leuven B-3001, Belgium; E-Mails: miguel.barrios@biw.kuleuven.be (M.B.); jamshid.farifteh@biw.kuleuven.be (J.F.); w.w.verstraeten@tue.nl (W.W.V.); pol.coppin@biw.kuleuven.be (P.C.); 3 National Reference Laboratory for Hantavirus Infections, Laboratory of Clinical Virology, Rega Institute, KU Leuven, Minderbroedersstraat 10, Leuven B-3000, Belgium; E-Mails: piet.maes@rega.kuleuven.be (P.M.); jan.clement@uzleuven.be (J.C.); marc.vanranst@rega.kuleuven.be (M.V.R.); 4 Royal Netherlands Meteorological Institute (KNMI), Climate Observations, PO Box 201, De Bilt NL-3730 AE, The Netherlands; 5 Eindhoven University of Technology, Applied Physics, PO Box 513, Eindhoven 5600 MB, The Netherlands; 6 Evolutionary Ecology Group, University of Antwerp, Groenenborgerlaan 171, Antwerpen 2020, Belgium; E-Mail: katrien.tersago@ua.ac.be

**Keywords:** biogeography, bank voles, genetic algorithm, GARP, GIS

## Abstract

The bank vole (*Myodes glareolus*) is the natural host of *Puumala virus* (PUUV) in vast areas of Europe. PUUV is one of the hantaviruses which are transmitted to humans by infected rodents. PUUV causes a general mild form of hemorrhagic fever with renal syndrome (HFRS) called nephropathia epidemica (NE). Vector-borne and zoonotic diseases generally display clear spatial patterns due to different space-dependent factors. Land cover influences disease transmission by controlling both the spatial distribution of vectors or hosts, as well as by facilitating the human contact with them. In this study the use of ecological niche modelling (ENM) for predicting the geographical distribution of bank vole population on the basis of spatial climate information is tested. The Genetic Algorithm for Rule-set Prediction (GARP) is used to model the ecological niche of bank voles in Western Europe. The meteorological data, land cover types and geo-referenced points representing the locations of the bank voles (latitude/longitude) in the study area are used as the primary model input value. The predictive accuracy of the bank vole ecologic niche model was significant (training accuracy of 86%). The output of the GARP models based on the 50% subsets of points used for testing the model showed an accuracy of 75%. Compared with random models, the probability of such high predictivity was low (χ^2^ tests, *p* < 10^−6^). As such, the GARP models were predictive and the used ecologic niche model indeed indicates the ecologic requirements of bank voles. This approach successfully identified the areas of infection risk across the study area. The result suggests that the niche modelling approach can be implemented in a next step towards the development of new tools for monitoring the bank vole’s population.

## Abbreviations

ENM: Ecological Niche Modelling
GARP: Genetic Algorithm for Rule-Set Prediction
GBIF: Global Biodiversity Information Facility
HFRS: hemorrhagic fever with renal syndrome
NE: mild form of HFRS called nephropathia epidemica

## 1. Introduction

Fifteen emerging zoonotic or vector-borne infections with increasing impact on humans in Europe were identified during the period 2000–2006. Global climate change may be a major contributor to the spread of these zoonotic diseases [1,2]. Rodent borne hantavirus infections are part of this list [2]. Puumala virus (PUUV), hosted by the bank vole (*Myodes glareolus*), is such a hantavirus. It is common over vast areas of Europe and causes a general mild form of HFRS called nephropathia epidemica (NE) [3].

Human hantavirus epidemics have often been explained by bank vole abundance [4,5]. Climate can influence host defence, vectors, pathogen and habitat [1]. Although studies of the effects of local climatic variability have been important in revealing the influences of weather condition on plant phenology, it is demonstrated that large-scale climatic variability can also influence the phenology of plants (e.g., [6]).

In Belgium few studies have explored the environmental correlates of hantavirus transmission. The study of [7] showed that the spatial distribution of the PUUV infection is associated with the combination of factors linked to the vector and host population, to human behaviour and to landscape characteristics. They have concluded that a large part of the spatial variation in disease risk can be explained by environmental factors which control the host and virus ecology as well as human behaviour.

Furthermore, several studies showed the link between the abundance of bank vole’s food and bank vole’s population dynamics and NE incidence. Clear evidence for this can be found in the mast year phenomenon, which is defined as the abnormal abundance of seed production by oak or beech trees [5,8,9,10]. It is generally believed that rodent peak populations occur after the mast years and cause NE outbreaks in Western Europe [9,10,11]. Since vegetation characteristics are an important mechanism for understanding and predicting NE outbreaks [12], advanced new vegetation monitoring technology, such as remote sensing by satellites, can now offer valuable tools for mapping NE outbreaks. For example, it has recently been shown that during the 2001–2007 observation periods, a significant increase in the length of the forest growing season could be estimated by using remote sensing data. This increase was most pronounced in densely forested areas and has been linked with NE endemic areas [12]. Furthermore NE cases have been described by data-based modelling approach as a function of the inputs: average measured monthly precipitation (mm) and temperature (°C) in Belgium, as well as the carrying capacity (vole·ha^−1^) estimated by the model of [13] over an 11 years study period (1996–2008) [14]. The observed changes in vole population density and PUUV incidence in Europe illustrate how climate change can influence the outbreaks of hantaviruses through effects on the voles population [15].

Accepting the fact that the environmental factors play a major role in hantavirus transmission in Western Europe, the ecological niche modelling (ENM) technique is used in this study to assess suites of environmental factors that identify potential areas at risk due to occurrence of hosts. It is hypothesized that spatial variation of climate variables can be used to develop a predictive model that estimates geographical distribution of bank voles. The output of this study will assist the public health officials to visualize the spatial distribution of bank vole populations and forecast where future changes in host suitability may occur. This approach can be used as a tool to advise the public where to avoid visits to forests.

## 2. Materials and Methods

### 2.1. Study Area

Bank vole population are spread in three different biotopes in Europe namely taiga, tundra and temperate broadleaf forests. The most favourable biotope of bank voles are the temperate forests of Western and Central Europe and Boreal forest in Fenno-Scandia. West European rodent population dynamics are mainly affected by food resources and less by other mechanisms. In the boreal zones (e.g., Finland), the primarily coniferous forests do not show clear masting events and the vole cycles are determined predominantly by interactions between voles and their specialist mammalian predators and winter food resources [16,17].

Similar dynamic patterns were observed in the bank vole populations and the NE cases in Belgium, North of France and East of Germany. Previous research showed the link between the abundance of the bank vole’s food on the bank vole’s population dynamics and NE incidence (e.g., [5,9,10,18]). Clear evidence for this can be found in the mast year phenomenon. It is generally believed that rodent peak populations occur after the mast years and cause NE outbreaks in Belgium, North of France (along the Belgian border) and Germany (e.g., [9,10]). In 2005 the number of NE cases increased unexpectedly in Belgium, Germany and of France, with 372, 387, 253 NE cases, respectively [10]. As far as the temperate broadleaf forests are concerned, and from 1992 on, we noted that most NE cases occurred in the Ardennes (forested South of Belgium), on both sides of the Franco-Belgian border around the river Meus, and particularly in very limited areas wherein beechnuts were abundant, and not so much acorns [11]. That is areas with a dense coverage of the same deciduous broad-leaf tree species, the European beech (*Fagus sylvatica*) seemed to predispose to an abundance of local bank voles, and consequently to outbreaks of NE [19]. Later on, with increased assessment of the spread of NE in temperate Europe, it became clear that the West-European regions with the highest endemicity of NE, were exactly corresponding to the regions with a dense beech tree coverage, *i.e.*, not only the French and Belgian Ardennes, but also the whole North-East of France, and the South of Germany [20]. Therefore, we narrowed our study to Belgium, Switzerland, West of Germany, North of France, North of Italy and West of Austria.

### 2.2. Point Occurrence Information

The spatial distribution for the bank vole’s occurrence data (latitude/longitude) were obtained from multiple sources. In total 46 bank vole’s occurrence points were used in this study to build the model. The main source of the occurrence records from 1885–2005 were obtained from the Global Biodiversity Information Facility (GBIF, http://data.gbif.org). There is a concerted global effort to digitize biodiversity occurrence data from herbarium and museum collections that together offer an unparalleled archive of life on Earth over the past few centuries. Since 2004 GBIF has provided a single point of access to specimen data from databases of biological surveys and collections.

**Figure 1:**
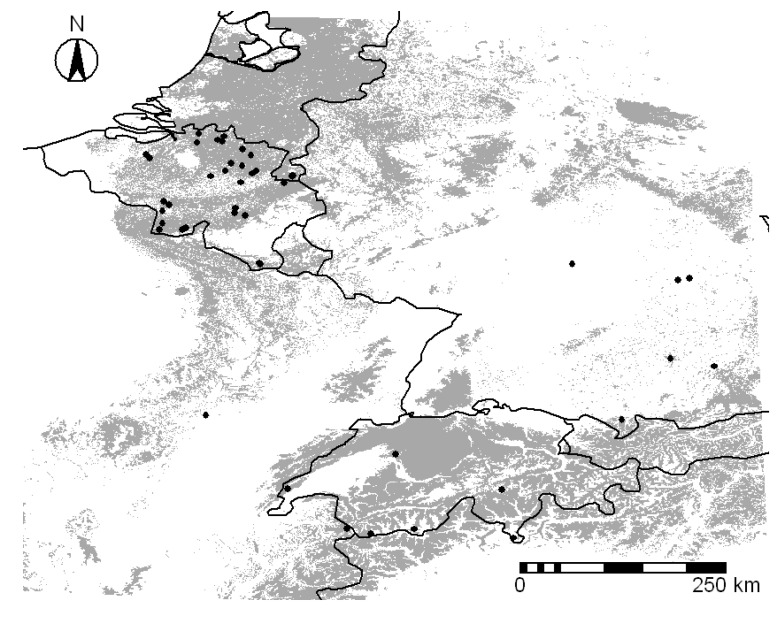
Map of geographical distribution of bank voles. Areas predicted to be habitable place for bank voles are indicated in grey. The dots represent the bank voles occurrence data points.

In Belgium the bank voles’ occurrence data points were obtained from the work of [7]. In addition we trapped bank voles in two different sites in Belgium (2009). The trapping sites were located in the municipalities of Gierle (Antwerp, Flanders) and Chimay (Henegouwen, Wallonia). The bank voles’ occurrence data points and the studied area are illustrated in Figure 1.

### 2.3. Environmental Data Layer

To model the ecological niche of the bank voles in the study area we used 23 environmental data layers. Typical environmental data sets used for ENM include topography (elevation, aspect, slope), climatology (temperature, precipitation), and biology (vegetation, other species distributions) [21].

The climatic and topographic data layers used in this study were extracted from the [22]. WorldClim is a set of global climate layers (climate grids) with a spatial resolution of 1 km^2^ [22].

Bioclimatic variables were derived from monthly temperature and rainfall values in order to generate more biologically meaningful variables. These are often used in ecological niche modelling (e.g., Maxent, GARP). The bioclimatic variables represent annual trends (e.g., mean annual temperature, annual precipitation) seasonality (e.g., annual range in temperature and precipitation) and extreme or limiting environmental factors (e.g., temperature of the coldest and warmest month, and precipitation of the wettest and driest quarters). The current climate data are interpolations of observed data, representative of 1950–2000.

The 23 environmental data layers used for the ecological niche modelling (ENM) included: topographic (elevation, aspect, slope), climatic data (Annual mean temperature, mean diurnal range (mean of monthly (max temp-min temp)), isothermality (mean diurnal range/temperature annual range ×100), temperature seasonality (standard deviation ×100), max temperature of warmest month, min temperature of coldest month, temperature annual range, mean temperature of wettest quarter , mean temperature of driest Quarter, mean temperature of warmest quarter, mean temperature of coldest quarter, Annual precipitation, precipitation of wettest month, precipitation of driest month, precipitation seasonality, precipitation of wettest quarter, precipitation of driest quarter, precipitation of warmest quarter and precipitation of coldest quarter) and land cover data layers.

Land cover is the major driver of bank voles’ distributions (e.g., [7,23]). We used the 250 m resolution European Land Cover map provided by the CORINE programme (European Environment Agency, 2000).

### 2.4. GARP Modelling

GARP, developed by [21], is an iterative, artificial-intelligence-based approach to ENM [24]. It employs a “superset” of rules to identify the ecological niche of a species [21].

After building an initial set of rules, GARP makes iterative changes to them: in each iteration (“generation”), rules undergo “genetic” changes insertions, deletions, point mutations, and crossing over among rules can all occur. In this manner, GARP explores solution space flexibly to identify relationships between environmental conditions and species’ occurrences. For each iteration the predictive accuracy of the model is evaluated based on the testing data set randomly sampled from the whole study area [25]. The rule is accepted and incorporated into the model if the change in predictive accuracy increases; otherwise, the rule is rejected and dropped. A one-tailed chi-square test on the difference between the probability of the predicted value before and after the employment of the rule was used to determine the statistical significance of these rules [21]. The final rule set is obtained at when iterative changes fail to improve the model’s predictive accuracy, or when the algorithm runs 1,000 iterations [21].

Predictive accuracy of the model defined using the expression: (*a* + *b*)/(*a* + *b* + *c* + *d*) Where a is the number of points where the model predicted present and the input point (actual point) was a presence record, b is the number of points where the model predicted absence and the input point was an absence record, c is the number of points where the model predicted absence and the input point was a presence record; and d is the number of points that the model predicted present but no information is available about the species present at that locations [26].

A commission error (*b*/(*b* + *d*)) occurs when the model predicts a species to occur where it does not (*i.e.*, including areas not actually inhabited); an omission error (*c*/(*a* + *c*)) occurs when the model fails to predict a species occurrence where it does in fact occur (*i.e.*, excluding areas actually inhabited) [26].

Another use of GARP is to point out what environmental factors are more significant or important than others for a given species. This operation is called environmental layer Jack-knifing [21]. By running multiple models with combinations of layers, either all combinations or a subset of all combinations with a fixed number of layers, the user can analyze the results of the experiment using multiple linear regressions to check which layers have a significant impact on the resulting errors [21].

GARP divides randomly the species occurrence data (model input points) into training and testing data sets, the former used to build the model and the latter used to validate the model accuracy. Presampling the data to even proportions allows consistent comparison of accuracy between species and produces more reliable models [21]. Thus the bank voles’ occurrence points were divided into training (50%) and testing (50%) sub data sets. All 23 environmental data layers were used by running multiple tasks with different combination of layers.

To analyze each layer individually and to determine the existence of a significant positive or negative correlation with the commission and omission errors we used multiple linear regression model. Independent variables for a multiple linear regression analysis indicated whether the specific layer was used on the task or not (0 or 1). These values could be used to predict commission and omission errors as dependent variables. 

To choose the best subset models we ran the GARP with all combinations of selected significant layers. We eliminated all the models that had none zero omission error based on the independent test points. Then, one multiple linear regression model was developed as using the commission error as an outcome and the present or absent (0 or 1) of the particular environmental layer as a predictors (University of Kansas Center for Research, 2002).

The regression analysis was performed in Matlab® version 7.6 (R2008a) by using the stepwise regression function. Variables with the *p* values >0.05 were excluded from the model. 

The next step was to prepare and run another experiment using just those layers that are known to be significant on the model generation, minimize omission, and keep commission down to the species potential distribution in the study region (in this study we assumed that the potential distribution area of the bank voles are the natural grass land, broad leaves forest and mixed forest which consists of 30% of our study area).

A final ENM of bank voles was performed using only the significant environmental data layers identified in the Jack-knifing procedure. To choose the best subset models we ran the GARP with all combination of selected significant layers. For these final runs, the input bank voles’ occurrence data sets were divided into training (50%) and testing (50%) sub-data sets, and 20 model runs were performed as it was done in the work of [27].

We used the procedure of [26] for choosing the best subset of models. The procedure was based on the observation that: (1) models vary in quality; (2) variation among models involves an inverse relationship between errors of omission (leaving out true distributional area) and commission (including areas not actually inhabited); and (3) best models are clustered in a region of minimum omission and potential distribution area predicted [26]. To choose the best subsets of models, we: (1) eliminated all models that had non-zero omission error based on independent test points [28]; (2) calculated the potential distribution area predicted present among these zero-omission points; and (3) identified the best model closest to the overall potential optimal area predicted.

## 3. Results

### 3.1. Environmental Data Layer Jack-Knifing

The Jack-knifing method identified the slope, maximum temperature of the warmest month, temperature annual range, mean temperature of the coldest quarter, precipitation of warmest quarter, mean temperature of warmest quarter, mean diurnal temperature range, min temperature of the coldest month and CORINE land cover as significant (*p* < 0.025) predictive variables for modelling the ecological niche of the bank voles and had small commission and omission errors. 

### 3.2. Ecological Niche Modelling Predictions

The next step was to run the GARP with all combination of selected significant layers, using just those layers that were known to be significant for the model. We used the procedure of [26] for choosing the best model. The best model was built with only five of the selected significant environmental layers (Corine land cover, maximum temperature of the warmest month, temperature annual range, mean temperature of the coldest quarter, precipitation of warmest quarter). The best model of the geographical distribution of the bank voles is shown in Figure 1.

The output of the GARP models representing spatial distribution of bank voles were statistically significant (χ^2^ tests, *p* < 10^−6^), which indicates that, the models were quite predictive. The output of the GARP models based on the 50% subsets of points used for testing the model showed an accuracy of 75% and the training accuracy of the GARP model was 86%. This demonstrates that, the used ecologic niche model indeed presents the ecologic requirements of the bank voles.

To permit the visualisation of ecological niche variations we combined the input environmental grid and the final prediction of the geographical distribution of the bank voles to create a new grid that has a distinct value for each unique combination of environments. To make a visualisation more feasible we developed scatter plots to show the impact of the significant environmental factors and the ecological niche variation of the bank voles.

**Figure 2:**
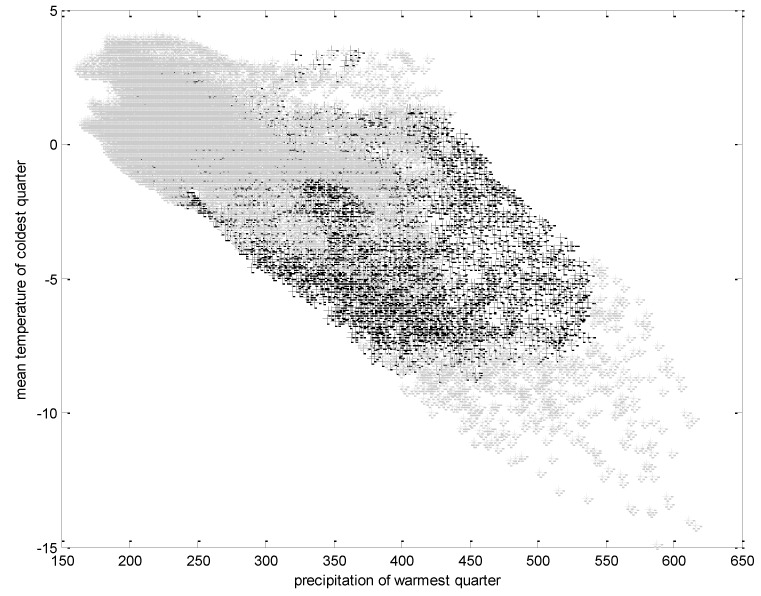
Modelled result of the ecological niche of the bank voles according to the precipitation of the warmest quarter (mm) and mean temperature of the coldest quarter (°C). The black crosses represent the suitable ecological niche of the bank voles.

**Figure 3:**
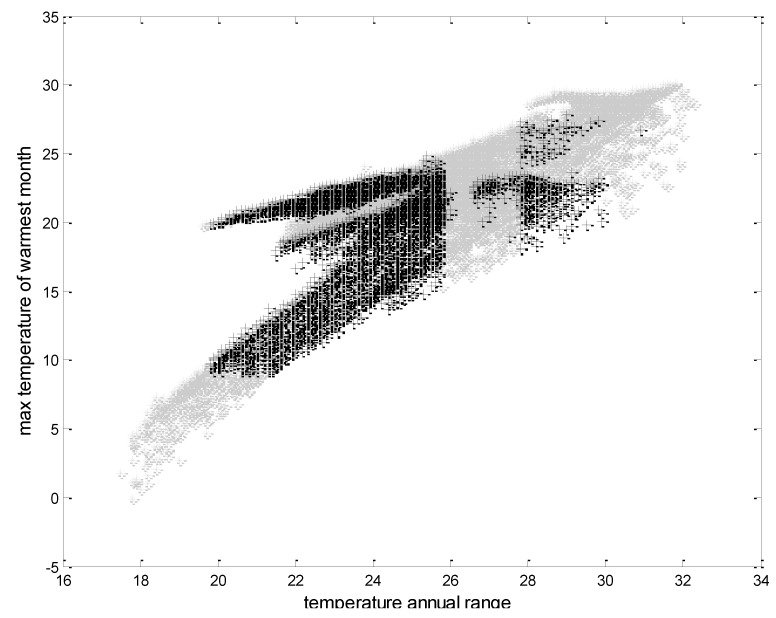
Modelled result of the ecological niche of the bank voles according to the temperature annual range and maximum temperature of the warmest months (°C). The black crosses represent the suitable ecological niche of the bank voles.

Bank voles were distributed in regions in which the precipitation of the warmest quarter is 300–550 mm, and in areas in which the mean temperature of the coldest quarter is between −5 and −10 °C (Figure 2). Furthermore, the area should have a moderate summer (the max temperature of the warmest month is between 10–25 °C) with the temperature annual range between 20–30 °C (Figure 3).

**Figure 4:**
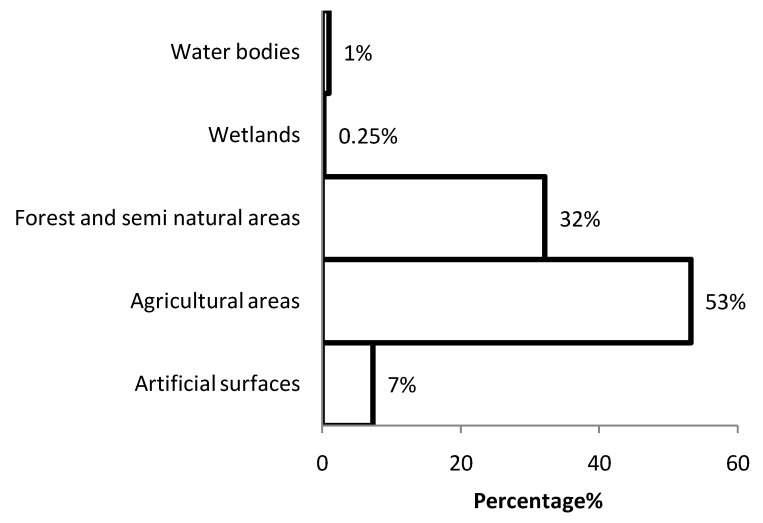
The percentage of the land cover in the study are based on the CORINE land cover.

**Figure 5:**
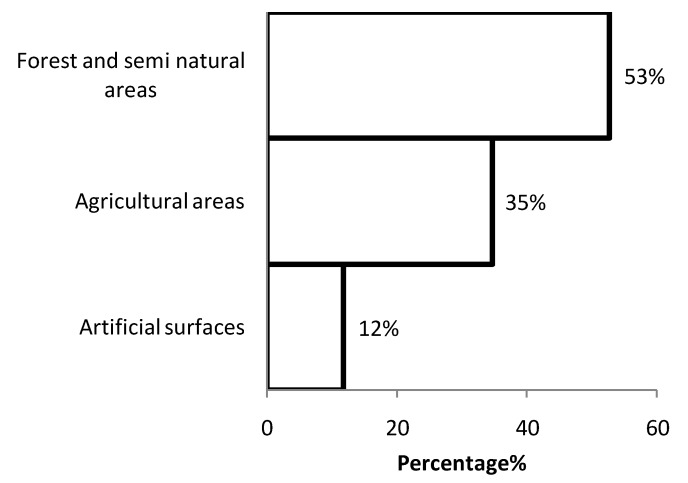
The distribution of the potential habitable areas of the bank voles based on the CORINE land cover category (percentage).

As we calculated the land cover fractions of the study area based on the CORINE land cover. The study area was mostly consisting of Agricultural (53%) and Forest and Semi-natural areas (32%). Artificial surfaces consisted only 8% of the study area (Figure 4) and Wetlands and Water Bodies together consist around 1% of the whole study area. In total 21% of the study area could be considered as a habitable place of which more than half of that area was covered with forests and semi natural areas (11% of the hole study area), around 35% of the potential geographical distribution of the bank voles was situated in agricultural area and around 10% of the potential geographical distribution of the bank voles was located in areas covered by artificial surfaces (Figure 5).

## 4. Discussion

The number of new NE cases is proportional to the number of susceptible humans who are at risk (e.g., people who have occupational activities related to forest work or farmers) [29]. The agricultural area covered half of the study area and one third of the agricultural surfaces are habitable for the bank voles. 87% of the habitable agricultural areas consist of pastures and land cover of complex cultivation patterns. The second largest land cover area in our study is the forest and semi natural areas. Our results show that 70% of the habitable areas for the bank voles is located in the forest (broad-leaved forest, coniferous forest and mixed forest) and natural grasslands. Only 12% of the habitable regions for the bank voles in our study area consist of artificial surface, of which 84% of the habitable place for the bank voles in this category is located in the discontinues urban fabrics. But a higher disease risk is expected in the land covers where the contact between humans and voles is more frequent.

Our results showed that in Western Europe bank voles live mainly in regions with moderate summer temperature and with a precipitation of 300–500 mm. The geographical distribution characteristics and seasonal variations of the bank voles population is indicating that bank vole habitat preference is linked with the climate factors such as temperature and precipitation. Thus climate variability can potentially affect the bank vole’s geographical distribution.

Under a clear set of assumptions, the GARP modelling approach allows indicating the area potentially used by a species. However, few species actually occupy all areas of potential distribution [30]. Some such areas indicated by the model as areas of potential habitable by a given species may be occupied by closely related species, or may represent suitable areas to which the species has failed to disperse [26].

PUUV can be transmitted directly by physical contact between infected bank voles and human or indirectly transmitted to humans by inhalation of air-suspended particles of urine, faeces, or saliva from infected bank voles [31,32]. Hence the transmission can only occur by the exposure of humans to the habitats of bank voles, although our results indicated that bank vole mainly occupy forest and semi natural areas. Control infection studies showed that humans are most likely to come into contact with rodents in rural locations close to the mixed pine and broad leaf forest [33]. Another study showed the importance of occupation activities related to the forest work; indicating the fact that most affected persons were male adults [34]. A serological survey for the prevalence of hantavirus infection in the Netherlands showed that among the individuals with a susceptible occupational risk (e.g., hunters, military, zoologists, veterinary *etc.*), animal trappers, forest workers, laboratory workers and farmers have the highest prevalence of hantavirus serum antibodies [35]. This confirms the existence of bank voles in agricultural areas. The survey further documented that the NE cases occur in eastern and southern rural and forest areas in the Netherlands [35]. As it was shown in our result, Myodes glareolus (bank vole) is commonly present in the Netherlands with exception of the islands of Goerre-Overflakkee, Vlieland and Ameland (D. Bekker, Zoogdiervereniging VZZ, pers. comm.).

The study of [36] quantified the relationship between sample size and the predictive accuracy of the GARP models. In that study they examined the GARP performance on predicting the special distribution of 103 species in Mexico (with the area of around 2 million km^2^) of which more than 200 occurrence records were available. They selected randomly the occurrence points for developing the predictive GARP model with different sample sizes. Their results indicated that 10–30 occurrence points are generally sufficient to achieve maximum predictive accuracy for a given species. Therefore 46 bank vole’s occurrence point seems to be more than enough to achieve the accurate modelling result in our studying area (with our study area of around 580,000 km^2^).

The objectives of this paper were to (1) point out the most significant or important environmental factors for the distribution of bank voles and (2) to model the ecological niche of bank voles. In order to get accurate results, despite the bias in the occurrence data points of bank voles (*i.e.*, with most of the samples being from Belgium), we limited the study area to the most endemic region in the Western Europe, having the same biotype and the same dynamic mechanisms underlying the bank vole population. This approach minimized the bias in the modelling results, as the relationship between the environmental factors and the habitat of the bank voles could be different on a larger scale compared to the study area.

The occurrence data of the bank voles are not homogeneous distributed over the study area with most of the samples being from Belgium. However it does not appear to be leading to a significant bias in the model (see Figure 1). The model predicts extensive habitats in the Netherlands, which confirms the presence of bank voles all over the Netherlands (D. Bekker, Zoogdiervereniging VZZ, pers. comm). To verify the accuracy of the predicted habitat area of the bank voles we compared them with the spatial distribution of the NE cases, taking into account that in Europe the geographical distribution of bank voles is wider than the distribution of NE [37]. France and Germany both represent endemic and disease free areas. The main HFRS endemic areas in France are in the Ardennes in Northern France, bordering with Belgium. The Ardennes region accounts for 30–40% of France NE cases. The department of Jura bordering on Switzerland is also an epidemic region in France [38]. In West Germany the Federal state most affected are Nordrhein Westfalen (bordering with Belgium and Netherlands) and Baden-Wurttemberg (bordering with Switzerland, which account for 45–80% of the German NE cases [38,39]. Our results confirmed that the consistent prediction of areas in France and Germany quite distant from the few sampled locations in those countries are not significantly affected by sampling bias and that the predictive habitable areas for bank voles overlap with the endemic and epidemic regions in our study area.

A limitation of this study that should be acknowledged is that it is based on the occurrence data of bank voles from 1885–2005 and that the average climatology data are interpolations of observed data, representative for 1950–2000. The GARP model managed to identify regions that are potential habitats of bank voles in long periods but it is unable to show the dynamics of spatial distribution of the bank voles. Therefore, it would be preferable to have the data in real time by trapping campaigns to examine the impact of the climate change on the spatial distributions of the bank voles and movement of the bank vole’s populations from one place to another. The land cover changes can be another source of the bias in our modeling result because the habitat of the bank voles could have been changed since the collection of the bank vole occurrence data. Therefore, a niche model with more recent occurrence points might be needed to validate our results. 

Trapping bank voles is an expensive and time consuming activity. Another approach to reduce the cost of the survey would be to combine patients’ data (e.g., most probable region that people became infected based on the control case studies) with the trapping data to detect the infection on a finer scale and in different time periods of low and high infection rate. For example [40] used the ecological niche modelling approach to identify the areas of hantavirus transmission risk in forested areas in Brazil based on the human case occurrence and not on the reservoir host species.

The density of bank voles’ population and resulting NE cases can show a dramatic increase in response to ecological conditions of previous years (e.g., [10]). The improvements of environmental conditions are followed by an immediate increase in the population of susceptible bank voles, and with a slight delay (from one to four months) the outbreak of human infections also increases [14,29]. Thus, yearly monitoring of the significant environmental factors which control the spatial distribution of bank voles (such as vegetation coverage and weather data) might provide us with an expert tool to prevent the NE outbreaks. Updating the land cover maps, the climatic (temperature, precipitation), and biological (vegetation, other species distributions) maps together with studying the existence of the bank voles in different regions, may be used to monitor, model and predict the spatial distribution of the bank vole populations and thus potential occurrence of NE cases in our study area, 

## 5. Conclusions

In this study we managed to produce the map of potential geographical distribution of bank voles in Western Europe based on the occurrence data points of bank voles and climate and land cover maps. We identified the most significant environmental factors to build the ecological niche model of bank voles namely: the slope, maximum temperature of the warmest month, temperature annual range, mean temperature of the coldest quarter, precipitation of warmest quarter, mean temperature of warmest quarter, mean diurnal temperature range, min temperature of the coldest month and CORINE land cover.

The output of the GARP models representing spatial distribution of bank voles was significant (training accuracy of 86%). The output of the GARP models based on the 50% subsets of points used for testing the model showed an accuracy of 75%. Random models show that, the probability of such predictivity is very unlikely (χ^2^ tests, *p* < 10^−6^). This suggests that the GARP models were quite predictive and that the used ecologic niche model indeed indicates the ecologic requirements of bank voles.

Our results confirmed that the changes that affect the geographical distribution of bank voles is climate related. Our result showed that local temperature and precipitation affects habitat suitability for bank voles in Western Europe. The niche modeling could help the public health authorities to identify potential habitat areas of bank voles with possible transmission risk to humans. It can also identify the significant environmental factors which are involved in defining the habitable area of the species, based on environmental conditions. Visualisation of ecological niches provided insights into the ecological basis of the bank voles’ population spatial distribution. When we assume that the relationship between the environmental conditions and bank voles’ habitat is not changing, we can predict the geographical distribution of the bank voles under different climate scenarios. 
